# Neurenteric Cyst: Case Report and Operative Video

**DOI:** 10.7759/cureus.8714

**Published:** 2020-06-20

**Authors:** Sricharan Gopakumar, Nisha Gadgil, Malcolm F McDonald, Ron Gadot, Alexander E Ropper

**Affiliations:** 1 Neurological Surgery, Baylor College of Medicine, Houston, USA

**Keywords:** neurenteric cyst

## Abstract

Neurenteric cysts are rare, congenital lesions of the spinal axis composed of endodermal tissue arising from poor segmentation of the notochord. A 36-year-old patient presented with arm paresthesias and incontinence with imaging revealing a lesion in the C6-C7 region most consistent with neurenteric cyst. After partial resection of the lesion, the patient regained all neurological function. Here, we provide a brief overview of this rare neuropathologic entity and demonstrate surgical resection of neurenteric cyst through operative video.

## Introduction

Neurenteric cysts are uncommon congenital spinal lesions which account for only 0.7%-1.3% of spinal axis tumors [1]. These cysts result from inappropriate segmentation of the notochord during embryogenesis causing endodermal tissue to remain in the spinal canal [1]. Neurenteric cysts present more commonly in males, cause focal sensorimotor symptoms at their spinal level, and typically appear as non-contrast-enhancing lesions that are T1 isointense and T2 hyperintense [2,3]. Gross total resection is the most common management [2-4]. We present a case of neurenteric cyst and highlight surgical management of this lesion through operative video.

## Case presentation

A 36-year-old male presented with worsening left arm and leg paresthesias and issues with bladder control. Symptoms were noted shortly after a hospitalization for bacterial meningitis two months prior. Initially, the patient felt tingling and numbness in the left posterior arm, medial forearm, and left hand involving the third, fourth, and fifth digits. These symptoms eventually progressed to the left leg and were accompanied by episodes of urinary incontinence and ataxia.

**Figure 1:**
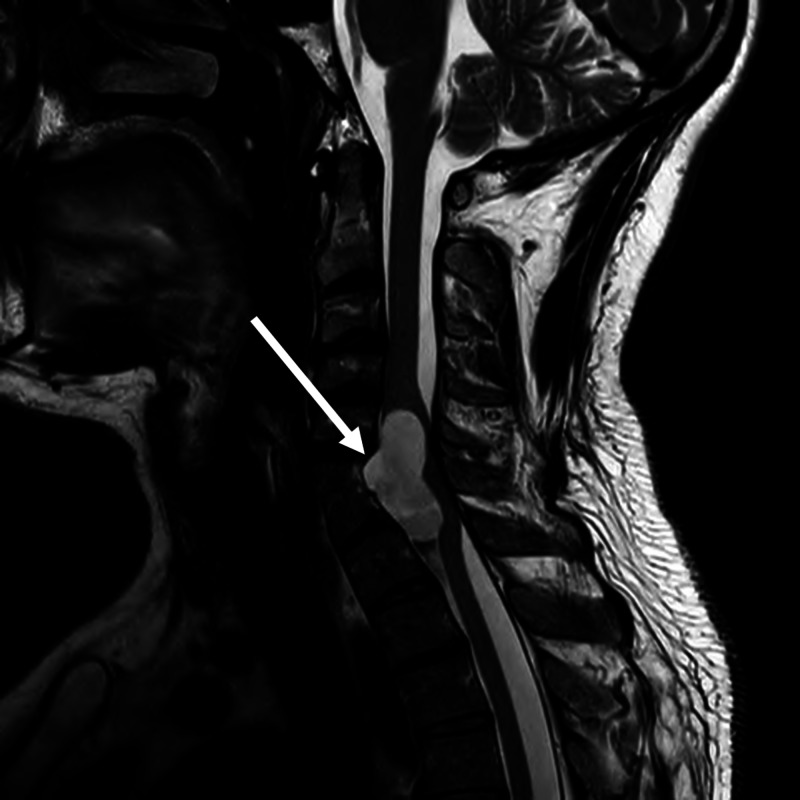
T2 MRI cervical spine demonstrating cystic lesion (white arrow) at C6/C7.

On physical exam, the patient had weakness of the left-hand intrinsic muscles (3/5), wrist extensors/flexors (4/5), and triceps (4/5); his other extremities had full strength. Sensation was diminished in the left C8 dermatome. Upper and lower extremity reflexes were normal, gait was normal, and Hoffman’s sign was negative. MRI cervical spine (Figure 1) revealed a large anterior intradural, extramedullary cystic spinal lesion at C6/C7 with compression of the spinal cord posteriorly. The lesion is T2 hyperintense and T1 hypointense and measures 1.6 x 2.7 x 3.5 cm in size. Also notable was fusion of the C6 and C7 vertebrae.

The lesion was most consistent with neurenteric cyst, though expanded differential could also include schwannoma, epidermoid cyst, and arachnoid cyst. Treatment of the cyst involved partial laminectomies at C5 and T1 and a C6-C7 laminectomy. This was followed by fenestration and excision of the cyst (Video 1) and a C6-C7 laminoplasty.

**Video 1:** Operative video of neurenteric cyst resection. Orientation: right = cranial, left = caudal

Careful opening of the arachnoid was performed with sharp dissection using microscissors. Gentle dissection down the right aspect of the spinal cord, in between the C6 and C7 nerve roots, allowed for identification of the cyst. The cyst was then opened sharply and a significant amount of fluid was evacuated. At the dural entry of the right C7 nerve root, a small amount of mucinous material was resected and sent for frozen section, which confirmed neurenteric cyst. Given the ventral location of the cyst under the spinal cord, complete resection of the entire cyst was not possible.

**Figure 2:**
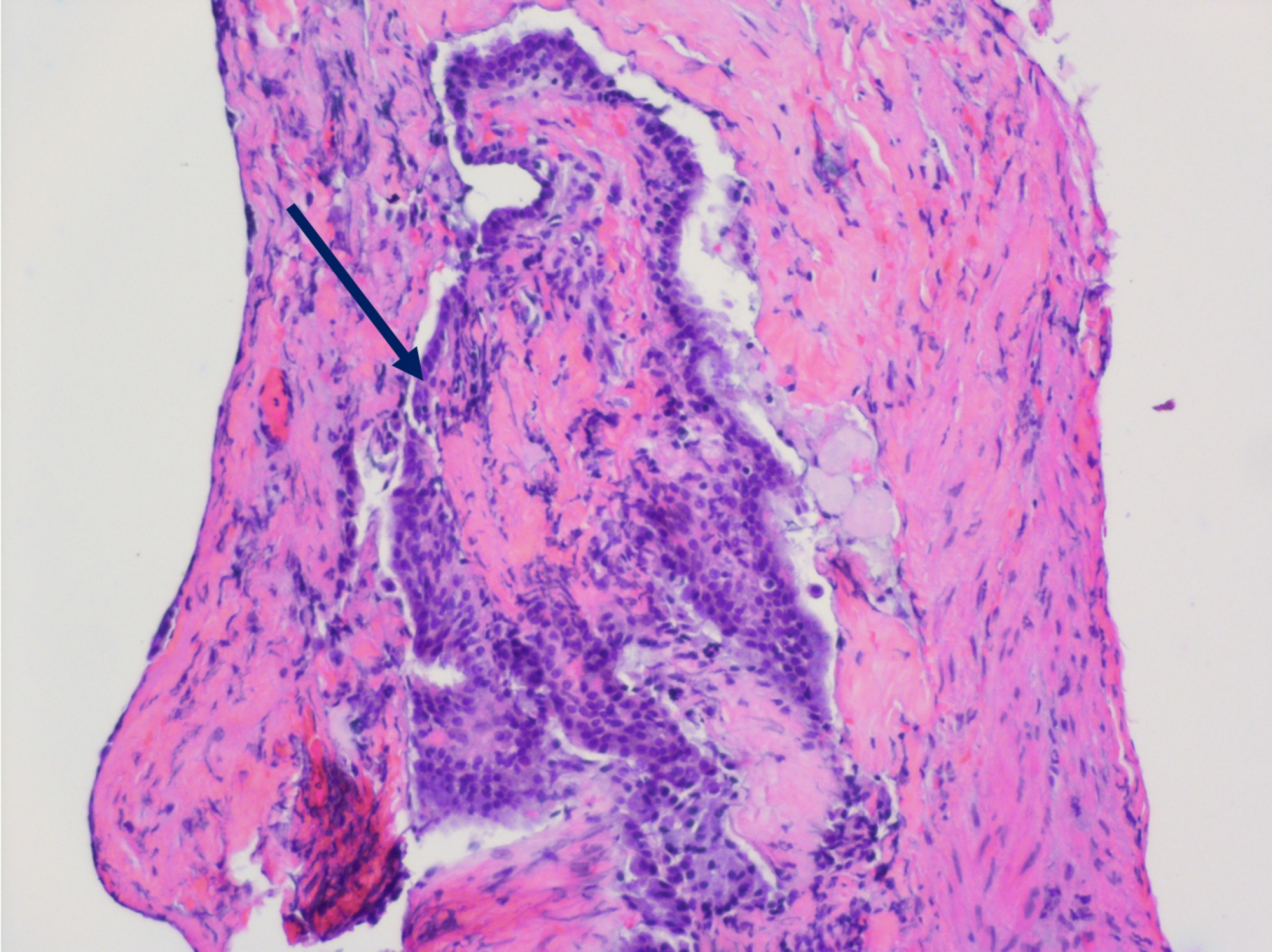
Mucin-producing simple columnar/cuboidal goblet cells (black arrow) on hematoxylin and eosin staining.

Postoperatively, the patient showed immediate improvement in left arm paresthesias and slight residual numbness in digits 3-5. The patient was discharged on postoperative day 3 and was ambulatory and voiding. Full strength was reached at two-week follow-up. On pathological analysis, the lesion demonstrated mucin-producing goblet cells surrounded by a central cystic cavity (Figure 2) and was formally diagnosed as a congenital, non-segmenting neurenteric cyst.

**Figure 3:**
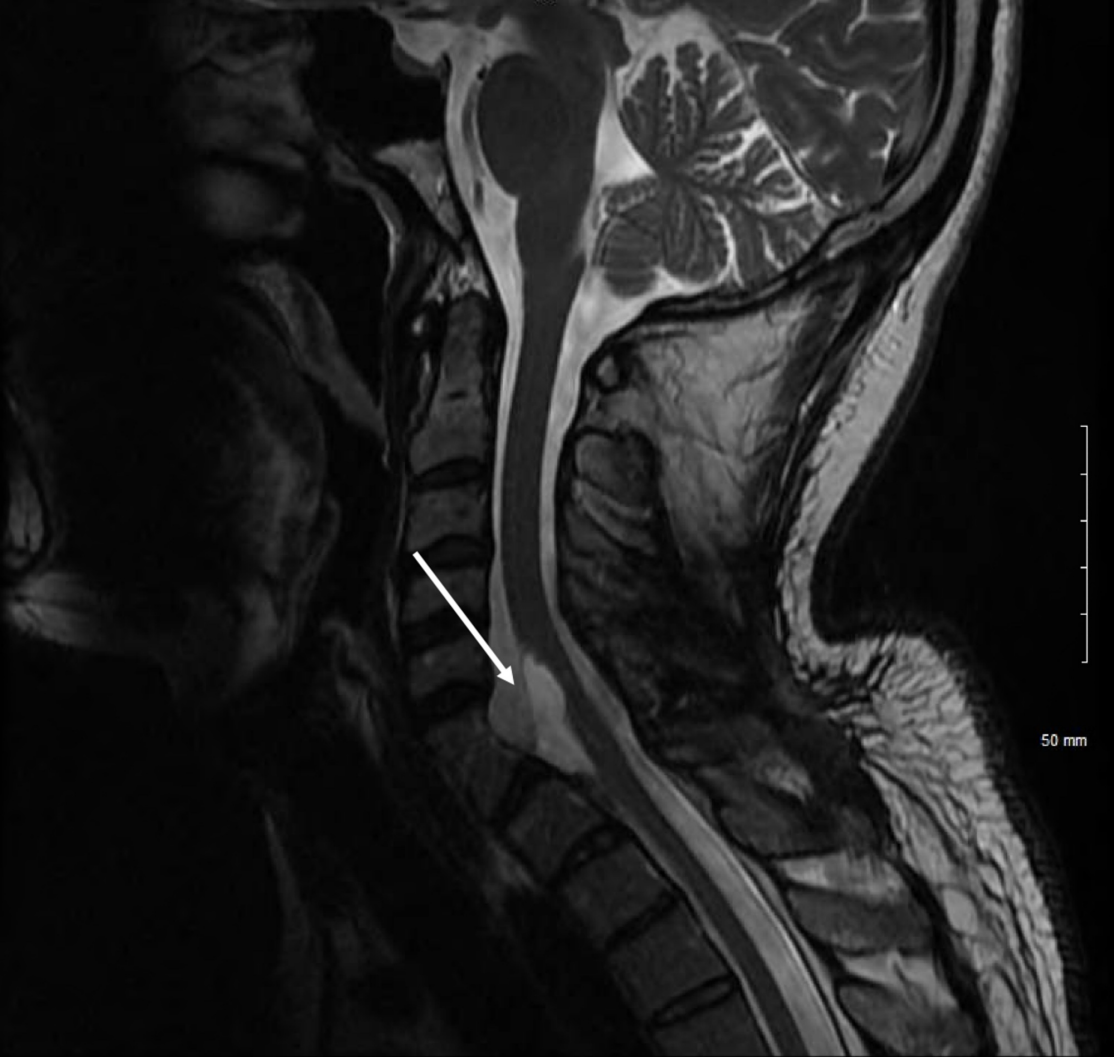
T2 MRI demonstrating residual cyst (white arrow) at six-month follow-up.

At six-month follow-up, the patient had complete resolution of left upper extremity dysesthesias, no residual weakness, and markedly improved numbness with residual numbness in only the distal fourth and fifth digits. Follow-up MRI (Figure 3) shows residual cystic lesion continuous with the anterior cervicothoracic spinal cord at C5-T1, measuring 9.5 mm x 6.5 mm x 31 mm (previously 16 mm x 27 mm x 35 mm).

## Discussion

Neurenteric cysts are rare, congenital lesions that are usually ventral to the spinal cord in the intradural, extramedullary compartment of the cervical and thoracic regions of the spinal canal [1]. Composed of heterotopic endodermal tissue that results from incomplete separation of the notochord and endoderm during the third week of embryogenesis, neurenteric cysts eventually present during the second or third decade of life, as did our patient, and are more common in males [1]. Consistent with our patient’s presentation, most common symptoms are pain, radiculopathy, or myelopathy, but symptoms may wax and wane [1]. In 50% of cases, other bony abnormalities of the spine such as spinal dysraphism, scoliosis, spina bifida, split cord malformation, or Klippel-Feil syndrome can be observed [1,3,4].

Radiographically, neurenteric cysts are typically hypo- or isointense, non-enhancing lesions on T1 MRI and hyperintense on T2 MRI [2]. Histologically, neurenteric cysts are composed of a central cystic cavity surrounded by mucin-producing simple columnar or cuboidal goblet cells that can be ciliated or non-ciliated [5]. Surgical management is the recommended course of treatment, with a posterior approach the most widely reported technique used [1,2]. Gross total resection via a posterior approach is usually not possible, as in our case, with recurrences reported in the literature likely due to the ventral location of these lesions [1-4]. In general, post-surgical recurrence has been reported in up to 37% of cases [1,6,7]. In a case series of 23 patients, partial excision had higher recurrence rates but shared similar clinical outcomes due to the benign nature and favorable prognosis of this entity [2]. In a series of 121 spinal neurenteric cysts, gross total resection was possible in 44.6%, recurrence occurred in 22.7%, and progression free survival rate at 10 years was 66.2% [8]. Ultimately, surgical intervention allows for resolution of neurological symptoms, and though gross total resection may be desired, subtotal resection allows for similar outcomes.

## Conclusions

A 36-year-old patient presenting with arm paresthesias and incontinence was found to have a C6-C7 neurenteric cyst. We demonstrate partial surgical resection of this rare congenital tumor located in the ventral spinal cord in an accompanying surgical video.
